# Clusters of Circulating let-7 Family Tumor Suppressors Are Associated with Clinical Characteristics of Chronic Hepatitis C

**DOI:** 10.3390/ijms21144945

**Published:** 2020-07-13

**Authors:** Yi-Shan Tsai, Ming-Lun Yeh, Pei-Chien Tsai, Ching-I Huang, Chung-Feng Huang, Meng-Hsuan Hsieh, Ta-Wei Liu, Yi-Hung Lin, Po-Cheng Liang, Zu-Yau Lin, Shinn-Cherng Chen, Jee-Fu Huang, Wan-Long Chuang, Chia-Yen Dai, Ming-Lung Yu

**Affiliations:** 1 Hepatobiliary Division, Department of Internal Medicine and Hepatitis Center, Kaohsiung Medical University Hospital, Kaohsiung Medical University, Kaohsiung 807, Taiwan; 1016ys@gmail.com (Y.-S.T.); yeh_ming_lun@yahoo.com.tw (M.-L.Y.); pctsai1225@gmail.com (P.-C.T.); tom65222@gmail.com (C.-I.H.); fengcheerup@gmail.com (C.-F.H.); hsmonyan@gmail.com (M.-H.H.); davyliu@gmail.com (T.-W.L.); 990076@kmuh.org.tw (Y.-H.L.); pocheng.liang@gmail.com (P.-C.L.); zuyali@kmu.edu.tw (Z.-Y.L.); chshch@kmu.edu.tw (S.-C.C.); jf71218@gmail.com (J.-F.H.); waloch@kmu.edu.tw (W.-L.C.); fish6069@gmail.com (M.-L.Y.); 2 Health Management Center, Kaohsiung Medical University Hospital, Kaohsiung Medical University, Kaohsiung 807, Taiwan; 3 Department of Occupational Medicine, Kaohsiung Medical University Hospital, Kaohsiung Medical University, Kaohsiung 807, Taiwan; 4 Faculty of Internal Medicine, College of Medicine, Graduate Institute of Clinical Medicine, Kaohsiung Medical University, Kaohsiung 807, Taiwan; 5 Lipid Science and Aging Research Center (LSARC), Kaohsiung Medical University, Kaohsiung 807, Taiwan; 6 Center for Infectious Disease and Cancer Research, Kaohsiung Medical University, Kaohsiung 807, Taiwan; 7 Department of Biological Science and Technology, College of Biological Science and Technology, National Chiao Tung University, Hsin-Chu 300, Taiwan

**Keywords:** chronic hepatitis C, let-7 family, hepatitis C virus, miRNA, biomarker, hepatocellular carcinoma

## Abstract

Hepatitis C virus (HCV) infections can cause permanent liver-related diseases, including hepatocellular carcinoma (HCC). Low mortality and incidence of HCC have been observed in patients with chronic hepatitis C undergoing direct-acting antiviral therapy. Tumor suppressive let-7 family members are down-regulated in HCC. The present study, therefore, aimed to investigate whether expression levels for the full spectrum of let-7 family members (let-7a, 7b, 7c, 7d, 7e, 7f, 7g, 7i, and miR-98) in the circulatory system are useful as surveillance biomarkers for liver-related diseases to monitor treatment efficacy during HCV infection. To this end, we measured the levels of mature circulating let-7 family members using quantitative reverse transcription-PCR in 236 patients with HCV infection, and 147 age- and sex-matched controls. Using hierarchical cluster analysis and principal component analysis, three clusters were obtained after measuring expression levels of let-7 family members in the patients and controls. Cluster 1 included let-7a/d/e/g, Cluster 2 comprised let-7b and let-7i, and Cluster 3 comprised let-7c/f/miR-98. Let-7b/c/g represented the three clusters and showed the best survival response to liver cancer when analyzed with respect to patient data. Therefore, considering the circulating levels of let7 b/c/g as representatives of the let-7 family may facilitate effective monitoring of liver-related disease.

## 1. Introduction

Chronic hepatitis C (CHC) virus infection can cause serious liver disorders. It can also increase the risk of hepatocellular carcinoma (HCC) and the progression of severe hepatic and extrahepatic diseases [1]. Compared to non-virological response (NVR), the sustained virological response (SVR) resulting from treatment with pegylated interferon-α (PegIFNα) and ribavirin (RBV) is associated with a lower risk of HCC development [2]. However, patients with significant hepatic fibrosis remain at high risk for HCC, even when they achieve SVR with antiviral therapy [3,4]. Since 2013, the development of direct-acting antivirals (DAAs) has increased SVR rates by 95% above those of interferon (IFN)-based treatments in patients with chronic HCV infection regardless of viral genotype [5]. However, there is no evidence that HCC occurrence or recurrence differs between patients receiving DAA or IFN therapy [6]. Furthermore, the annual post-SVR HCC incidence (approximately 1%) remains higher than that for cancers of other organs. Therefore, it is necessary to establish a clinical strategy for monitoring cancer risk in post-SVR patients [7,8].

Mature microRNAs (miRNAs) are short, single-stranded RNA molecules (approximately 22 nucleotides in length) that post-transcriptionally silence gene expression and play important roles in a broad variety of biological processes, including intrinsic antiviral immunity. Chen et al. systematically characterized serum/plasma miRNAs and found that they were stable, reproducible, and consistent among individuals of the same species. These miRNAs also represent promising, non-invasive biomarkers for diagnosing cancer and other diseases [9]. Furthermore, it has been suggested that miRNAs can be used as a prediction model for the treatment outcome of HCV virus genotype 1 infection [10].

Previous studies have shown that the miRNA let-7b exhibits a significant anti-HCV effect [11], and that IFNα rapidly modulates the expression of let-7s with anti-HCV activity by targeting *IGF2BP* [12]. In our previous study, we demonstrated the effects of let-7g on HCV infection in vitro in clinical tissue and serum samples. We found that IFN/RBV treatment induces let-7g expression. Furthermore, overexpression of let-7g reduces the expression of the HCV gene and core protein level, thereby inhibiting viral replication. Let-7g and IFN/RBV treatment also synergistically inhibits HCV replication and represses Lin28A/B [13]. 

The let-7 family, comprised of ten members (let-7a, 7b, 7c, 7d, 7e, 7f, 7g, 7i, miR-98, and miR-202), target the 3′ untranslated regions (UTRs) of genes essential for development and are conserved from *Caenorhabditis elegans* to humans [14]. Next-generation sequencing and microarray studies have revealed that various HCC-specific miRNA signatures in the liver tissue showed lower let-7 (a/b/c/d/e/f/g) expression levels compared to healthy liver tissues [15]. Matsuura et al. performed a longitudinal miRNA microarray study on plasma and extracellular vesicles (EVs) in patients with CHC and found that the plasma levels of circulating let-7(a/c/d) were higher than those in EVs, and were inversely correlated with the severity of hepatic fibrosis [16]. 

Despite these studies, the roles of mature let-7 family members (let-7a, 7b, 7c, 7d, 7e, 7f, 7g, 7i, and miR-98) in the circulating plasma of patients with CHC and their clinical relevance remain unclear. Moreover, due to the large number of let-7 family members, and the difficulty associated with obtaining liver tissues from CHC patients and the control group, detection of all family members for the purposes of monitoring liver-related diseases is not feasible. Nevertheless, this study aimed to evaluate the association between circulating let-7 family members and the clinical characteristics of CHC patients. We also examined the potential for use of nine mature let-7 family members as non-invasive biomarkers for CHC patients, using cluster analysis and principal component analysis (PCA). An independent dataset was then employed to evaluate the clusters of the let-7 family in normal tissue (N), and tumors (T) from the Cancer Genome Atlas (TCGA) liver cancer samples. Here we demonstrate that assessing the levels of the circulating let-7 family members could represent a promising new method to monitor liver-related diseases.

## 2. Results

### 2.1. Clinical Characteristics of Patients

The characteristics of the subjects enrolled in this study are listed in Table 1. Compared with the healthy control group, CHC patients had significantly higher aspartate transaminase (AST, *p* < 0.0001), alanine aminotransferase (ALT, *p* < 0.0001), gamma-glutamyl transferase (γGT, *p* < 0.0001), and fasting plasma glucose (*p* < 0.0001) levels. Alternatively, creatinine (Cr, *p* < 0.0001), white blood cell counts (WBC, *p* < 0.0001), platelet counts (PLT, *p* < 0.0001), hemoglobin (*p* < 0.0001), total triglycerides (TG, *p* = 0.0002), total cholesterol (CHLO, *p* < 0.0001), HDL cholesterol (HDL-C, *p* < 0.0001), and LDL cholesterol (LDL-C, *p* < 0.0001) were significantly lower in CHC subjects.

### 2.2. Circulating Let-7 Family Member Profiling 

**Figure 1:**
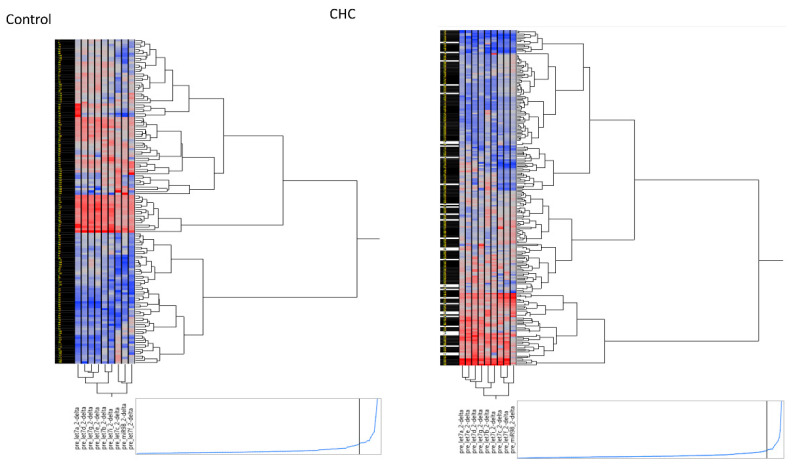
Hierarchical clustering plot (heatmap) of circulating let-7 family in the control group without hepatitis C virus (HCV) infection (*n* = 147) and HCV-infected patients (*n* = 236).

To determine the expression of let-7 family members in the blood, a qRT-PCR was performed. The −△*C*t (cycle threshold) value of each miRNA was measured and normalized to that of cel-39, as it showed the most consistent Ct value among all donors (27.10 ± 0.82 and 27.18 ± 1.06 for healthy controls and CHC patients, respectively) (Table S1). After statistical analyses, the expression levels of the let-7 family members were significantly lower in patients with CHC compared to the control group (Table 2 and Figure 1). The distribution of individuals is provided in Figure S2.

### 2.3. Three Clusters Distinguish the Let-7 Family Members 

Due to let-7 family members (let-7a, 7b, 7c, 7d, 7e, 7f, 7g, 7i, and miR-98) with highly correlated (Table S2). To further investigate associations between the expression levels of let-7 family members in individuals from the control and HCV-infected groups, hierarchical clustering was performed using Ward’s method. Three distinct clusters of let-7 family members were identified, in both the control and HCV-infected groups as represented in the hierarchical clustering plot (heatmap, Figure 1). Cluster 1 included let-7a, let-7d, let-7e, and let-7g. Cluster 2 was comprised of let-7b and let-7i, while Cluster 3 was made of let-7c, let-7f, and miR-98. We also performed a PCA to investigate the similarities between the expression levels of let-7 family members among the study participants and found that Cluster 3 (let-7c, f, and miR-98) was clearly distinct from clusters 1 and 2 in both patient groups (Figure S3).

### 2.4. Circulating Let-7 Family Expression Levels Correlate with Baseline Clinical Parameters 

Correlation analyses between differentially expressed let-7 family members and clinical parameters were performed, including characteristics such as age, indicators of liver damage or injury (AST and ALT), platelet count, and HCV viral load (Table 3). The expression levels of Cluster 1 members (let-7a and let-7g) were significantly negatively correlated with AST (*r* = −0.1898 and −0.2038; *p* = 0.0037 and 0.0018, respectively). Meanwhile, the expression of Cluster 1 members (let-7a/d/e/g) were significantly positively correlated with PLT (*r* = 0.2433, 0.2209, 0.2113, and 0.1911; *p* = 0.0002, 0.0007, 0.0012, and 0.0034, respectively). However, no correlation was observed between any of the three let-7 clusters and ALT or HCV load. These results demonstrate that Cluster 1 (let-7a/d/e/g) was a better indicator of clinical characteristics than the other two let-7 clusters. Additionally, the circulating let-7 levels were not correlated with the HCV genotype (Table S3) or HCV viral load (Table S4).

### 2.5. Let-7b/c/g Levels Are Associated with Clinical Progression

**Figure 2:**
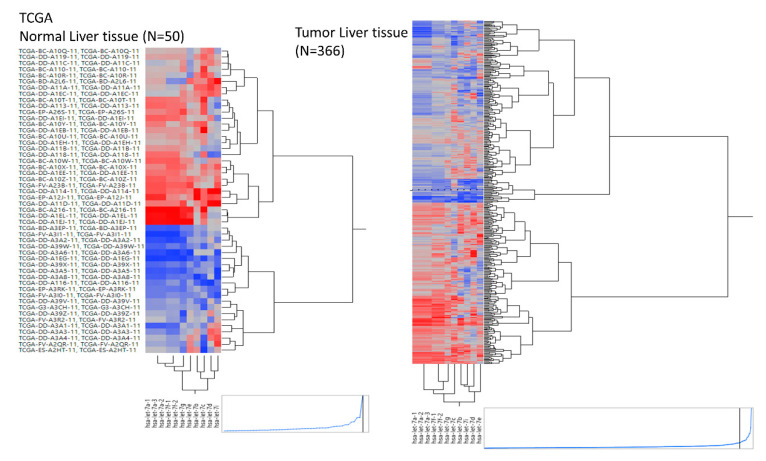
Hierarchical clustering plot (heatmap) of the let-7 miRNA family expression in healthy (*n* = 50) and tumor (*n* = 366) liver tissue samples obtained from TCGA-LIHC.

**Figure 3:**
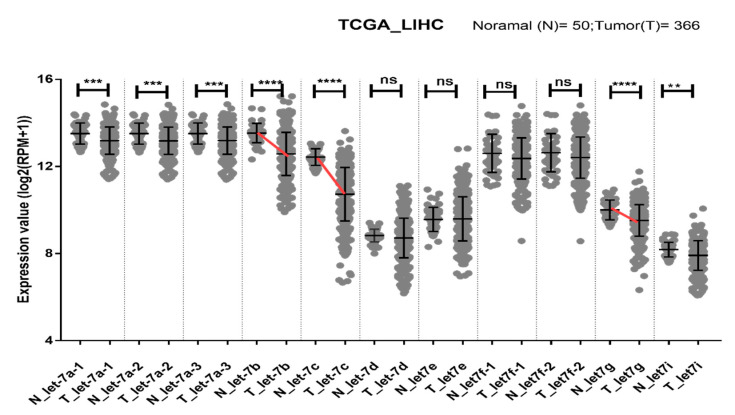
Dot plot showing the relationship between the expression of let-7 family members in normal (*n* = 50) and tumor (*n* = 366) tissues from liver cancer patients in the TCGA dataset. Log_2_ (RPM +1) transformed values for let-7 family members are shown as the mean ± SD. Prominent declines in let-7b/c/g are indicated by red lines. Statistical significance was assessed using the Mann–Whitney test. The *p* values are represented as follows: ** = *p* < 0.01, *** = *p* < 0.001, **** = *p* < 0.0001.

**Figure 4:**
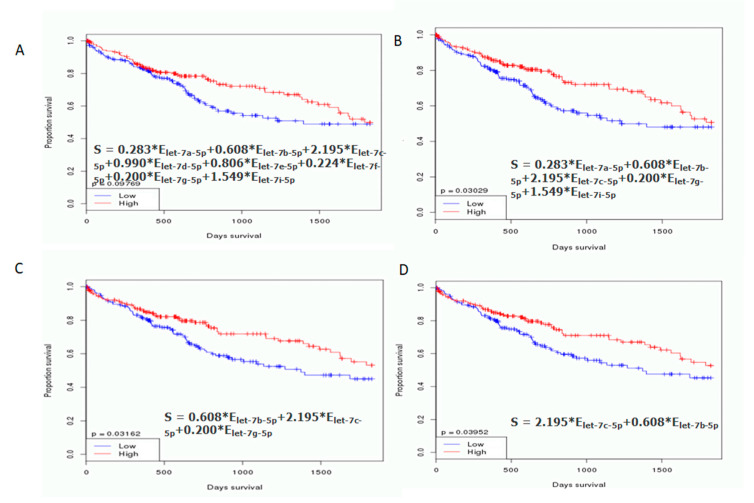
Kaplan–Meier (KM) survival analysis curve for let-7 family expression in TCGA-LIHC patients. The KM survival curves were examined according to (**A**) let-7 a/b/c/d/e/f/g/i expression level and (**B**) let-7 a/b/c/g/i expression levels. No differences in overall survival were observed. (**C**) let-7 b/c/g expression levels and (**D**) let-7 b/c expression levels show clear differences in overall survival. Statistical significance was obtained by log-rank tests, with α = 0.05.

An independent dataset was used to further evaluate the let-7 clusters in normal tissue (N) and tumors (T) from TCGA liver cancer samples. A hierarchical clustering plot revealed three clusters, grouped into let7a-1/a-2/a-3/f-1/f-2/g/e, let-7b/c, and let7d/i (Figure 2). The expression levels of let-7a/b/c/g/i were significantly lower in tumors than in healthy tissue. Furthermore, the greatest reduction in let-7b/c/g expression was observed in tumors (red line, Figure 3). This observation was supported by OncomiR (www.oncomir.org.), which was used to explore the associations between TCGA-LIHC (liver hepatocellular carcinoma) survival data and the let-7 family expression levels. Kaplan–Meier survival analysis demonstrated that patients with high expression of let-7b/c/g had significantly increased overall survival compared to patients with low expression (*p* = 0.03162, Figure 4). Taken together, these data indicate that reduced expression of let-7b/c/g may be associated with liver tumor progression.

## 3. Discussion

Circulating mature miRNAs are small RNAs measuring approximately 22 nucleotides, and are known to be stable in the serum/plasma [9]. These nucleic acids represent novel non-invasive biomarkers for liver inflammation, liver fibrosis, liver cancer [17], and non-alcoholic fatty liver disease (NAFLD), [18]; they have also been shown to be useful for cancer detection [19]. Next-generation sequencing or microarray methods may be useful for identifying let-7 (a/b/c/d/e/f/g) members that are down-regulated in plasma or liver tissues to diagnose patients with hepatic fibrosis or hepatocellular carcinoma [15,16]. The ten mature let-7 family members are derived from 13 precursors located in nine different chromosomes with similar seed regions [20]. These let-7 family members target 3′ UTRs of genes that are essential for development and have been conserved from *C. elegans* to humans [14]. However, the precise roles of circulating let-7 family members in humans remain uncharacterized. Here, we studied nine mature let-7 family members, including let-7a, 7b, 7c, 7d, 7e, 7f, 7g, 7i, and miR-98 (*Homo sapiens* [has]-miR-202 was excluded), in the circulating plasma of patients with CHC and healthy controls, using TaqMan quantitative RT-PCR assays. To the best of our knowledge, this is the first study to demonstrate that circulating let-7 family members can be classified into three similar clusters in control and CHC groups. The highest expression levels were found for Cluster 2 (let-7b and let-7i), while the lowest expression levels were found in Cluster 3 (let-7c/let-7f/miR-98). Moreover, the expression levels of circulating let-7 family members in patients with CHC were lower than those in the healthy population. Here we also identified the circulating let-7 b/c/g as representatives from each of the three let-7 clusters as the most effective markers for detecting liver-related diseases based on their strong association with clinical characteristics.

Our previous study showed that the expression level of let-7g in liver tissues was significantly lower in NVR patients than in SVR patients and that antiviral treatment with IFN/RBV could induce let-7g expression [13]. Moreover, the ectopic overexpression of let-7 family members was found to repress HCV core protein, and HCV loads in a cell model system [12,13]. However, in this study, the HCV load and HCV genotype were not found to be dependent factors and were negatively correlated with the expression levels of let-7 family members in the circulatory system. Therefore, let-7 family members might specifically target naked HCV RNA in liver tissue or cell models, but not HCV RNA within the envelope coat. 

Previously Kirschner et al. reported specific miRNAs for which the profiling suggested an influence of hemolysis (miR-16, -451, -92a, let-7b, -103, -106a, 17, -21, -210, -27a, -31,-625-3p, -92a) as well as miRNAs (let-7a and let-7d) that appeared to be unaffected by hemolysis [21]. We, therefore, also measured the absorbance peak at 414 nm to detect the level of hemolysis and observed values of 0.192 ± 0.1478 (mean ± SD) for the control group, and 0.3143 ± 0.1723 (mean ± SD) for the CHC group (*p* < 0.05; Figure S4). Hemolysis was higher in CHC group. Therefore, the circulating let-7 (a/d/e/g) Cluster1 for the control group and CHC group might be unaffected by hemolysis. 

Down-regulated let-7 miRNA expression in the circulatory system might result in an increase in interleukin-10 (IL-10) from CD4+ T-cells, providing the virus with an important survival advantage by manipulating the host immune response [22]. Early IL-10 elevation has been shown to strongly suppress the priming of naïve HCV–specific CD8+ T-cells, causing T-cell failure and viral persistence [23]. In addition, HCV induces the expression of toll-like receptor 4 (TLR4), enhancing the production of IFNβ and IL-6 [24]. Vespasiani-Gentilucci et al. confirmed that dominant TLR4 hyperexpression in patients with CHC was significantly correlated with the inflammatory score and degree of fibrosis, indicating that TLR4 plays an important role in the pathogenesis of HCV-related chronic liver diseases [25]. It has also been reported that let-7b can target TLR4 through 3′ UTR post-transcriptional regulation and attenuates NF-κB activity [26]. Furthermore, expression levels of circulating let-7 (a/c/d) are inversely correlated with the severity of hepatic fibrosis [16], indicating that decreases in expression levels of let-7 family members might be involved in HCV infection and may cause inflammation, which is clinically and epidemiologically linked to cancer. NF-κB has been shown to have a causative role in inflammation. Iliopoulos et al. previously showed that transient activation of Src oncoproteins could trigger an NF-κB-mediated inflammatory response that directly activates Lin28 transcription and rapidly reduces the miRNA levels of let-7 family members [27]. 

The HCV core protein has been shown to have oncogenic potential [28]. Ali et al. demonstrated that the expression of an HCV sub-genomic replicon in cultured cells could cause them to acquire cancer stem cell-like signatures, including the enhanced expression of Lin28 and other proteins [29]. More importantly, Lin28b has been shown as sufficient to drive liver cancer and necessary for cancer maintenance in murine models. Many human cancers exhibit deregulated let-7 expression [14,30], the specificity of which is inhibited by Lin28A/B [31]. 

Interestingly, we also observed that let-7 (a/d/e/g) expression levels were positively correlated with PLT. Platelets are known therapeutic targets for preventing ischemic damage, enhancing liver regeneration, and inhibiting hepatitis progression [32]. These miRNAs are also inversely correlated with AST and ALT, which are markers of liver damage. Together, these results indicate that decreases in let-7 b/c/g levels might be involved in HCV infection and damage; moreover, the literature suggests these could promote inflammatory responses and tumorigenesis associated with HCV-related fibrosis or HCC.

In human genomic loci clusters, miRNA genes, including let-7 genes, are frequently located at fragile sites. Inflammatory status promoters increase the production of reactive oxygen species, leading to oxidative DNA damage by reducing DNA repair and increasing genomic instability of these fragile sites [33]. Genomic regions involved in cancers as tumor suppressor genes, such as *let-7g/miR-135−1*, are located in fragile sites of the ARP-DRR1 region in 3p21.1-21.2. [34]. Therefore, decreasing levels of let-7 family members may not only be an important marker for disease progression, but may also suggest fragile site instability and promotion of oncogenes.

Certain limitations were noted in this study. First, plasma samples are easily contaminated with peripheral blood mononuclear cells. Although previous studies have reported that let-7b/c/g levels in PBMCs decline rapidly in HIV infection [22,35], HIV patients were excluded in the current study. Second, the relationship between expression levels of mature let-7 family members and inflammatory factors was unclear. Detailed analysis of other inflammatory mediators as intermediate variables is required. Such an analysis may provide additional information regarding the roles of other inflammatory mediators in the progression of HCV infection. Third, a large population-based follow-up study that includes additional circulating miRNAs is needed. This would allow for the investigation of baseline vs. post-antiviral treatment expression of the tumor suppressor let-7 b/c/g genes as potential early monitoring targets for patients who are at high risk for fibrosis, cirrhosis, and HCC. 

## 4. Materials and Methods 

### 4.1. Ethics Approval and Consent to Participate

This study was reviewed and approved by the IRB ethics committee (KMUHIRB-980176, KMUHIRB-20120097, and KMUHIRB-20140054 with the approval dates of 16 July 2009, 11 April 2012 and 7 November 2014, respectively). All protocols were approved by the ethical committee of the Kaohsiung Medical University Hospital, based on the International Conference on Harmonization for Good Clinical Practice. All participants provided written informed consent before enrollment.

### 4.2. Patient Cohort

We analyzed stored plasma extracted from the HCV patient database collected from one medical center and two regional core hospitals of the Kaohsiung Medical University between October 2009 and December 2016. The members of the age- and sex-matched control groups were enrolled from the same geographic communities. A total of 236 patients with HCV infection were enrolled in the HCV group. The following inclusion criteria were established before selection: i) Positive for anti-HCV antibodies for more than 6 months, and positive for HCV RNA by PCR assay; ii) negative for hepatitis B surface antigen (HBsAg) and no concomitant HIV; iii) negative for other types of hepatitis, including autoimmune hepatitis, primary biliary cirrhosis, sclerosing cholangitis, Wilson’s disease, and α_1_-antitrypsin deficiency; iv) daily ethanol consumption of <20 g (both females and males), as confirmed through an interview with the patient and a family member; and v) a high serum ALT level for 6 months preceding the study entry. The control group comprised 147 age- and sex-matched subjects without viral hepatitis who were selected based on the following inclusion criteria: i) Normal liver echogenicity, as determined by ultrasound sonography and ii) normal liver function test. The exclusion criteria for the control group included a current or past history of alcohol abuse (≥20 g ethanol per day), being pregnant, and seropositivity for HBsAg or anti-HCV antibody. All participants were advised to fast for 12 h overnight before the standard biochemistry tests, which included tests for Cr, AST, ALT, γGT, WBC, PLT, hemoglobin, TG, CHLO, HDL-C, LDL-C, AC_sugar, and HbA1c. Anthropometric data, including body weight and height, were obtained using standardized techniques. HBsAg was detected with commercially available enzyme-linked immunosorbent assay kits (Abbott Laboratories, North Chicago, IL, USA). HCV RNA and HCV genotype were assayed using a real-time PCR assay (RealTime HCV; Abbott Molecular, Des Plaines, IL, USA; detection limit, 12 IU/mL) [36]. 

### 4.3. MicroRNA Extraction from Plasma

A fixed volume of 200 μL of plasma was extracted using 1 mL Trizol LS reagent (Thermo Scientific, Wilmington, DE, USA), according to the manufacturer’s protocol. Molecular biology-grade 1–bromo–3–chloropropane (BCP; 300 μL/1 mL TRIzol LS) was then added. After centrifuging for 15 min at 12,000× *g* and 4 °C, a fixed volume of the aqueous phase was transferred into a new tube, which was treated with 2 µL of 100-pmol/L synthetic *C. elegans* Cel-39 as a spike-in control, and 10 µg of RNase-free glycogen as a co-precipitant carrier. The aqueous sample was mixed thoroughly with 100% molecular-grade isopropanol and incubated on ice for 1 h. A gel-like pellet was obtained after centrifugation at 12,000× *g* for 15 min. The flow-through was discarded and the pellet was washed with 80% ethanol. The pellet was again centrifuged at 7500× *g* for 5 min at 4 °C, and the supernatant was discarded. The RNA (including the miRNA) was air dried, eluted in 15 μL of RNase-free H_2_O and stored at −80 °C until further analysis. A total of 2 µL of each sample was used to measure the absorbance peak at 414 nm for the level of hemolysis on a NanoDrop™ 2000 (Thermo Fisher Scientific, Inc., Waltham, MA, USA) [37].

### 4.4. Quantification of Circulating miRNAs 

The total RNA (20 ng) was used as a template for reverse transcription, which was performed using the TaqMan MicroRNA Reverse Transcription kit and the associated miRNA-specific stem-loop primers to convert miRNA to cDNA as step 1 (Thermo Scientific, Wilmington, DE, USA), according to the manufacturer’s instructions. The *C. elegans* synthetic cel-miR-39-3p, a suitable and reproducible normalizer, was used as the spiked-in control [38]. The relative levels of individual miRNAs were amplified using the TaqMan^®^ Universal PCR Master Mix II, without uracil N-glycosylase (UNG), on a 7900HT Sequence detection system. Specific amplification was performed using the following program: 95 °C for 10 min, and amplification followed by 40 cycles of 95 °C for 15 s and 60 °C for 1 min as step 2. Specific primers of mature miRNA sequences were used for the TaqMan R microRNA assays (assay ID): hsa-let-7a (000377), hsa-let-7b (002619), hsa-let-7c (000379), hsa-let-7d (002283), hsa-let-7e (002406), hsa-let-7f (000382), hsa-let-7g (002282), hsa-let-7i (002221), hsa-miR-98 (000577), and an internal control, cel-miR-39-3p (000200). The hsa-miR-202 (002362) primer was excluded as it was under-determined in 99% of the samples with different nucleotides of the mature let-7 family members (Figure S1). The relative expression level of each let-7 member was determined using the comparative C_t_ method, which was defined as 2^−Δ*C*t^, where Δ*C*_t_ = *C*_t_ of the let-7 member − *C*_t_ of cel-39. 

### 4.5. miRNA-seq and Clinical Data from UCSC Xena Platform 

LIHC samples, including healthy (*n* = 50) and primary tumor tissues (*n* = 366) were obtained from the UCSC Xena platform. This platform provides interactive online visualization of cancer genomics datasets, such as TCGA, a public data resource [39]. Expression of the let-7 miRNA family mature strand was transformed according to RNA sequencing guidelines (Illumina Hiseq 2000) and presented as log_2_ (RPM +1). Kaplan–Meier survival curves and log-rank methods for the let-7 family clusters in LIHC were performed using OncomiR to evaluate overall survival (OS) rate [40].

### 4.6. Statistical Analysis

Statistically significant differences between the expression levels of let-7 family members in the different groups were determined using the Mann–Whitney test with Bonferroni correction for multiple comparisons. Pearson’s correlation analysis was used to assess the relationships between the let-7 family members and the different clinical parameters. Hierarchical clustering and PCA were performed to identify the distinguishable let-7 family members. All analyses were performed using JMP 12.0 (SAS Institute, Cary, NC, USA). Graphs were generated using the GraphPad Prism 5.0 software (San Diego, CA, USA).

## Figures and Tables

**Table 1:** Descriptive characteristics of baseline factors in study participants (*n* = 383) ^1^.

	Control Group (*n* = 147)	CHC Group (*n* = 236)	*p* Value
Age (years)	54.82 ± 9.96	55.11 ± 8.70	0.7705
Gender (F/M)	71/76	120/116	0.6276
BMI	25.09 ± 3.80	25.33 ± 3.57	0.5337
Total viral load [log_10_ (IU/mL) ^#^]	N/A	5.62 ± 0.84	N/A
HCV genotype (type1/non-type1/missing)	N/A	(131/100/5)	N/A
HCC (−/+) %	N/A	(94.9/5.1)	N/A
LC (−/+) %	N/A	(86/14)	N/A
Cr	0.98 ± 0.18	0.83 ± 0.25	< 0.0001 *
AST (IU/L)	24.62 ± 9.13	90.13 ± 52.90	< 0.0001 *
ALT (IU/L)	26.48 ± 18.23	122.48 ± 69.00	< 0.0001 *
GGT (IU/L)	33.08 ± 23.50	68.46 ± 65.51	< 0.0001 *
WBC	6370.88 ± 1699.14	5430.42 ± 1623.39	< 0.0001 *
PLT(×10^3^/µL)	252.85± 66.63	152.42 ± 61.06	< 0.0001 *
Hemoglobin (g/dL)	14.42 ± 1.44	14.02 ± 1.46	0.0085 *
TG (mg/dL)	133.42 ± 99.02	98.87 ± 56.52	0.0002 *
CHLO (mg/dL)	213.61 ± 39.74	169.11 ± 35.53	< 0.0001 *
HDL-C (mg/dL)	55.54 ± 14.26	45.47 ± 14.87	< 0.0001 *
LDL-C (mg/dL)	123.20 ± 33.33	99.54 ± 31.78	< 0.0001 *
AC_sugar (mg/dL)	92.26 ± 20.49	103.71 ± 32.25	< 0.0001 *
HbA1c (%)	5.88 ± 0.78	5.92 ± 1.07	0.7101

^1^ BMI, body mass index; HCC, hepatocellular carcinoma; LC, liver cirrhosis; Cr, creatinine; AST, aspartate aminotransferase; ALT, alanine aminotransferase; γGT, gamma-glutamyl transferase; WBC, white blood cell count; PLT, platelet count; TG, triglyceride; CHLO, cholesterol; HDL-C, HDL cholesterol; LDL-C, LDL cholesterol; AC_Sugar, fasting plasma glucose; HbA1c, hemoglobin A1c. All values are expressed as the mean ± standard deviation (SD). The *p* value was calculated for the continuous variables using the Student’s *t*-test or Mann–Whitney test, and the χ^2^ test was used for the categorical variables, with α = 0.05. * = *p* < 0.05. ^#^ The HCV virus loads were determined by log-transformation.

**Table 2:** Circulating let-7 family expression at baseline (Log_10_2^−^^△*C*t^) ^1^.

	Control (*n* = 147)	CHC (*n* = 236)	*p* Value
let-7a	−1.04 ± 0.74	−1.91 ± 0.57	< 0.0001 *
let-7b	−0.42 ± 0.43	−1.56 ± 0.60	< 0.0001 *
let-7c	−2.04 ± 0.36	−2.17 ± 0.44	< 0.0014 *
let-7d	−1.09 ± 0.61	−1.92 ± 0.50	< 0.0001 *
let-7e	−0.95 ± 0.64	−1.68 ± 0.62	< 0.0001 *
let-7f	−1.59 ± 0.54	−2.18 ± 0.40	< 0.0001 *
let-7g	−1.28 ± 0.52	−1.88 ± 0.55	< 0.0001 *
let-7i	−0.78 ± 0.47	−1.82 ± 0.57	< 0.0001 *
miR-98	−1.77 ± 0.54	−2.29 ± 0.33	< 0.0001 *

^1^ Data is presented as the mean ± SD. △*C*t = C_target_ – CT_cel39_; * = *p* < 0.05 following statistical analysis using an ANOVA with Bonferroni correction (α = 0.0056).

**Table 3:** Correlation between circulating expression of let-7 family members and various clinical parameters (*n* = 236) ^1^.

		Age	AST	ALT	PLT	HCV RNA
Cluster1	let7a	−0.0986	−0.1898 *	−0.0772	0.2433 *	0.1118
	let7d	−0.0846	−0.1757	−0.0747	0.2209 *	0.0829
	let7e	−0.1431	−0.1504	−0.0546	0.2113 *	0.1211
	let7g	−0.1495	−0.2038 *	−0.1041	0.1911 *	0.1386
Cluster2	let7b	−0.0172	−0.0746	−0.0106	0.1384	0.1118
	let7i	−0.1141	−0.126	−0.0153	0.1365	0.0848
Cluster3	let7c	−0.0456	−0.1298	−0.0448	0.0473	0.0753
	let7f	−0.045	−0.1576	−0.0499	0.0728	0.1442
	miR98	−0.0195	−0.0915	−0.0373	0.0046	0.062

^1^ Data represents correlation between miRNA expression and the values of each clinical parameter, as reported in Table 1. Correlation was determined using Pearson’s test. AST, aspartate aminotransferase; ALT, alanine aminotransferase; PLT, platelet count; HCV, hepatitis C viral load. * = *p* < 0.05 following the Bonferroni correction (α = 0.0056).

## Supplementary Materials

Supplementary materials can be found at https://www.mdpi.com/1422-0067/21/14/4945/s1.

## Abbreviations

AC_sugar	Fasting plasma glucose
ALT	Alanine aminotransferase
AST	Aspartate transaminase
BCP	1–bromo–3–chloropropane
BMI	Body mass index
CHLO	Total cholesterol
CHC	Chronic hepatitis C virus infection
Cr	Creatinine
CT	Cycle threshold
DAA	Direct-acting antiviral
EV	Extracellular vesicle
γGT	Gamma-glutamyl transferase
HCC	Hepatocellular carcinoma
HCV	Hepatitis C virus
HBsAg	Hepatitis B surface antigen
HDL-C	HDL cholesterol
HIV	Human immunodeficiency virus
Hsa	*Homo spaiens*
IFN	Interferon
KM	Kaplan–Meier
LC	Liver cirrhosis
LDL-C	LDL cholesterol
LIHC	Liver hepatocellular carcinoma
miRNA	MicroRNA
NAFLD	Non-alcoholic fatty liver disease
NVR	Non-virological response
OS	Overall survival
PCA	Principal component analysis
PegIFNα	Pegylated interferon-α
PLT	Platelet count
RBV	Ribavirin
SD	Standard deviation
SVR	Sustained virologic response
TCGA	The Cancer Genome Atlas
TG	Total triglycerides
TLR4	Toll-like receptor 4
WBC	White blood cell
