# Supplementary material for: Clusters of Circulating let-7 Family Tumor Suppressors Are Associated with Clinical Characteristics of Chronic Hepatitis C
Source: Int J Mol Sci. 2020 Jul 13;21(14):4945. doi: 10.3390/ijms21144945 (PMC7404305; doi:10.3390/ijms21144945)
Supplement: Supplementary file 1 [file ijms-21-04945-s001.zip › Supplemental Figures and Tables/Let-7 Supplementary Table_correct.docx]

(supplementary data)

Table S1. The cycle threshold (Ct) value of circulating let-7 family at baseline

| Ct | Non-HBV/non-HCV control(NBNC)  (n=147) | HCV infected  Case (only -HCV)  (n=236) | *p* |
| --- | --- | --- | --- |
| cel-39(mean±SD) | 27.10±0.82 | 27.18±1.06 | 0.4182 |
| let-7a (mean±SD) | 30.56±2.73 | 33.53±1.98 | <0.0001* |
| let-7b (mean±SD) | 28.49±1.46 | 32.37±2.00 | <0.0001* |
| let-7c (mean±SD) | 33.87±1.20 | 34.39±1.13 | <0.0001* |
| let-7d (mean±SD) | 30.72±2.20 | 33.55±1.70 | <0.0001* |
| let-7e (mean±SD) | 30.27±2.37 | 32.76±2.15 | <0.0001* |
| let-7f (mean±SD) | 32.38±2.00 | 34.43±1.12 | <0.0001* |
| let-7g (mean±SD) | 31.35±1.96 | 33.42±1.85 | <0.0001* |
| let-7i (mean±SD) | 29.69±1.65 | 33.20±1.84 | <0.0001* |
| miR-98 (mean±SD) | 32.96±1.89 | 34.78±0.61 | <0.0001* |

Note: the significance level (*) of Bonferroni correction as post hoc t-tests is p<0.0056

| Variable | by Variable | Case (N=236) | | | | Control (N=147) | | | |
| --- | --- | --- | --- | --- | --- | --- | --- | --- | --- |
|  |  | r | Lower 95% | Upper 95% | *p* | r | Lower 95% | Upper 95% | *p* |
| let7b | let7a | 0.609 | 0.5212 | 0.6839 | <.0001 | 0.6348 | 0.5271 | 0.7225 | <.0001 |
| let7c | let7a | 0.6182 | 0.5319 | 0.6917 | <.0001 | 0.2701 | 0.1132 | 0.4139 | 0.0009 |
| let7c | let7b | 0.5963 | 0.5066 | 0.6732 | <.0001 | 0.632 | 0.5236 | 0.7202 | <.0001 |
| let7d | let7a | 0.8182 | 0.7707 | 0.8566 | <.0001 | 0.7924 | 0.7232 | 0.8458 | <.0001 |
| let7d | let7b | 0.6461 | 0.5645 | 0.7153 | <.0001 | 0.8195 | 0.7582 | 0.8664 | <.0001 |
| let7d | let7c | 0.648 | 0.5667 | 0.7168 | <.0001 | 0.4084 | 0.2639 | 0.5349 | <.0001 |
| let7e | let7a | 0.8403 | 0.7979 | 0.8744 | <.0001 | 0.8177 | 0.7558 | 0.8651 | <.0001 |
| let7e | let7b | 0.6412 | 0.5588 | 0.7112 | <.0001 | 0.733 | 0.648 | 0.8 | <.0001 |
| let7e | let7c | 0.5981 | 0.5087 | 0.6747 | <.0001 | 0.3302 | 0.1778 | 0.4671 | <.0001 |
| let7e | let7d | 0.7804 | 0.7245 | 0.826 | <.0001 | 0.8988 | 0.8624 | 0.926 | <.0001 |
| let7f | let7a | 0.6493 | 0.5682 | 0.7179 | <.0001 | 0.5588 | 0.4359 | 0.6612 | <.0001 |
| let7f | let7b | 0.5895 | 0.4987 | 0.6674 | <.0001 | 0.5654 | 0.4438 | 0.6667 | <.0001 |
| let7f | let7c | 0.8459 | 0.8049 | 0.8789 | <.0001 | 0.5322 | 0.4047 | 0.6393 | <.0001 |
| let7f | let7d | 0.7087 | 0.6383 | 0.7673 | <.0001 | 0.6501 | 0.5453 | 0.735 | <.0001 |
| let7f | let7e | 0.631 | 0.5468 | 0.7025 | <.0001 | 0.6089 | 0.4954 | 0.7019 | <.0001 |
| let7g | let7a | 0.743 | 0.6793 | 0.7955 | <.0001 | 0.8225 | 0.7621 | 0.8687 | <.0001 |
| let7g | let7b | 0.5754 | 0.4825 | 0.6554 | <.0001 | 0.7744 | 0.7003 | 0.832 | <.0001 |
| let7g | let7c | 0.6639 | 0.5853 | 0.7301 | <.0001 | 0.3741 | 0.2259 | 0.5054 | <.0001 |
| let7g | let7d | 0.7584 | 0.6978 | 0.8081 | <.0001 | 0.9178 | 0.8879 | 0.94 | <.0001 |
| let7g | let7e | 0.7504 | 0.6883 | 0.8016 | <.0001 | 0.9094 | 0.8766 | 0.9338 | <.0001 |
| let7g | let7f | 0.7035 | 0.6321 | 0.763 | <.0001 | 0.6833 | 0.5859 | 0.7613 | <.0001 |
| let7i | let7a | 0.6785 | 0.6025 | 0.7423 | <.0001 | 0.6437 | 0.5378 | 0.7295 | <.0001 |
| let7i | let7b | 0.6918 | 0.6183 | 0.7533 | <.0001 | 0.9043 | 0.8697 | 0.93 | <.0001 |
| let7i | let7c | 0.6504 | 0.5694 | 0.7188 | <.0001 | 0.5709 | 0.4507 | 0.6708 | <.0001 |
| let7i | let7d | 0.6979 | 0.6254 | 0.7584 | <.0001 | 0.8271 | 0.768 | 0.8722 | <.0001 |
| let7i | let7e | 0.6708 | 0.5934 | 0.7359 | <.0001 | 0.7547 | 0.6754 | 0.8168 | <.0001 |
| let7i | let7f | 0.67 | 0.5925 | 0.7352 | <.0001 | 0.5528 | 0.4288 | 0.6563 | <.0001 |
| let7i | let7g | 0.6632 | 0.5845 | 0.7296 | <.0001 | 0.809 | 0.7446 | 0.8585 | <.0001 |
| miR98 | let7a | 0.3621 | 0.245 | 0.4688 | <.0001 | 0.3195 | 0.1663 | 0.4578 | <.0001 |
| miR98 | let7b | 0.4361 | 0.3259 | 0.5347 | <.0001 | 0.5631 | 0.4415 | 0.6644 | <.0001 |
| miR98 | let7c | 0.7161 | 0.6471 | 0.7734 | <.0001 | 0.5922 | 0.4759 | 0.6881 | <.0001 |
| miR98 | let7d | 0.437 | 0.3269 | 0.5355 | <.0001 | 0.5025 | 0.3708 | 0.6144 | <.0001 |
| miR98 | let7e | 0.3406 | 0.2218 | 0.4494 | <.0001 | 0.42 | 0.2769 | 0.5448 | <.0001 |
| miR98 | let7f | 0.721 | 0.653 | 0.7775 | <.0001 | 0.5868 | 0.4691 | 0.6841 | <.0001 |
| miR98 | let7g | 0.4251 | 0.3137 | 0.525 | <.0001 | 0.4782 | 0.3429 | 0.5941 | <.0001 |
| miR98 | let7i | 0.4435 | 0.334 | 0.5411 | <.0001 | 0.592 | 0.4757 | 0.6879 | <.0001 |

Table S2. The correlation of let-7 family members

Table S3. Trends of circulating let-7 levels response to HCV type (missing N=5)

| Log _10_2^-△Ct | HCV type 1  (n=131) | HCV non-type1  (n=100) | p |
| --- | --- | --- | --- |
| Let7a | -1.91±0.53 | -1.91±0.62 | 0.9887 |
| Let7b | -1.61±0.56 | -1.51±0.63 | 0.2447 |
| Let7c | -2.18±0.45 | -2.17±0.41 | 0.7992 |
| Let7d | -1.94±0.48 | -1.89±0.53 | 0.5108 |
| Let7e | -1.74±0.57 | -1.61±0.67 | 0.1335 |
| Let7f | -2.20±0.41 | -2.16±0.40 | 0.4542 |
| Let7g | -1.85±0.53 | -1.92±0.57 | 0.3309 |
| Let7i | -1.82±0.54 | -1.82±0.58 | 0.8963 |
| miR-98 | -2.30±0.37 | -2.27±0.27 | 0.3807 |

Note: the significance level of Bonferroni correction as post hoc t-tests is p<0.0056

Table S4. Trends of circulating let-7 levels response to Vius Load

| Log _10_2^-△Ct | Virus load  <800 IU/ml  (N=132) | Virus load  >=800 IU/ml  (N=101) | p |
| --- | --- | --- | --- |
| Let7a | -1.96±0.57 | -1.84±0.56 | 0.0997 |
| Let7b | -1.60±0.58 | -1.51±0.59 | 0.2291 |
| Let7c | -2.18±0.39 | -2.15±0.48 | 0.5460 |
| Let7d | -1.94±0.49 | -1.89±0.51 | 0.1368 |
| Let7e | -1.75±0.59 | -1.58±0.63 | 0.0361 |
| Let7f | -2.21±0.39 | -2.15±0.43 | 0.2441 |
| Let7g | -1.93±0.54 | -1.81±0.56 | 0.0966 |
| Let7i | -1.85±0.50 | -1.78±0.62 | 0.3553 |
| miR-98 | -2.27±0.34 | -2.30±0.33 | 0.5509 |

Note: the significance level of Bonferroni correction as post hoc t-tests is p<0.0056
