# Supplementary material for: Clusters of Circulating let-7 Family Tumor Suppressors Are Associated with Clinical Characteristics of Chronic Hepatitis C
Source: Int J Mol Sci. 2020 Jul 13;21(14):4945. doi: 10.3390/ijms21144945 (PMC7404305; doi:10.3390/ijms21144945)
Supplement: Supplementary file 1 [file ijms-21-04945-s001.zip › Supplemental Figures and Tables/Let7_Figures_Supplemental_202006.pptx]

## Slide 1
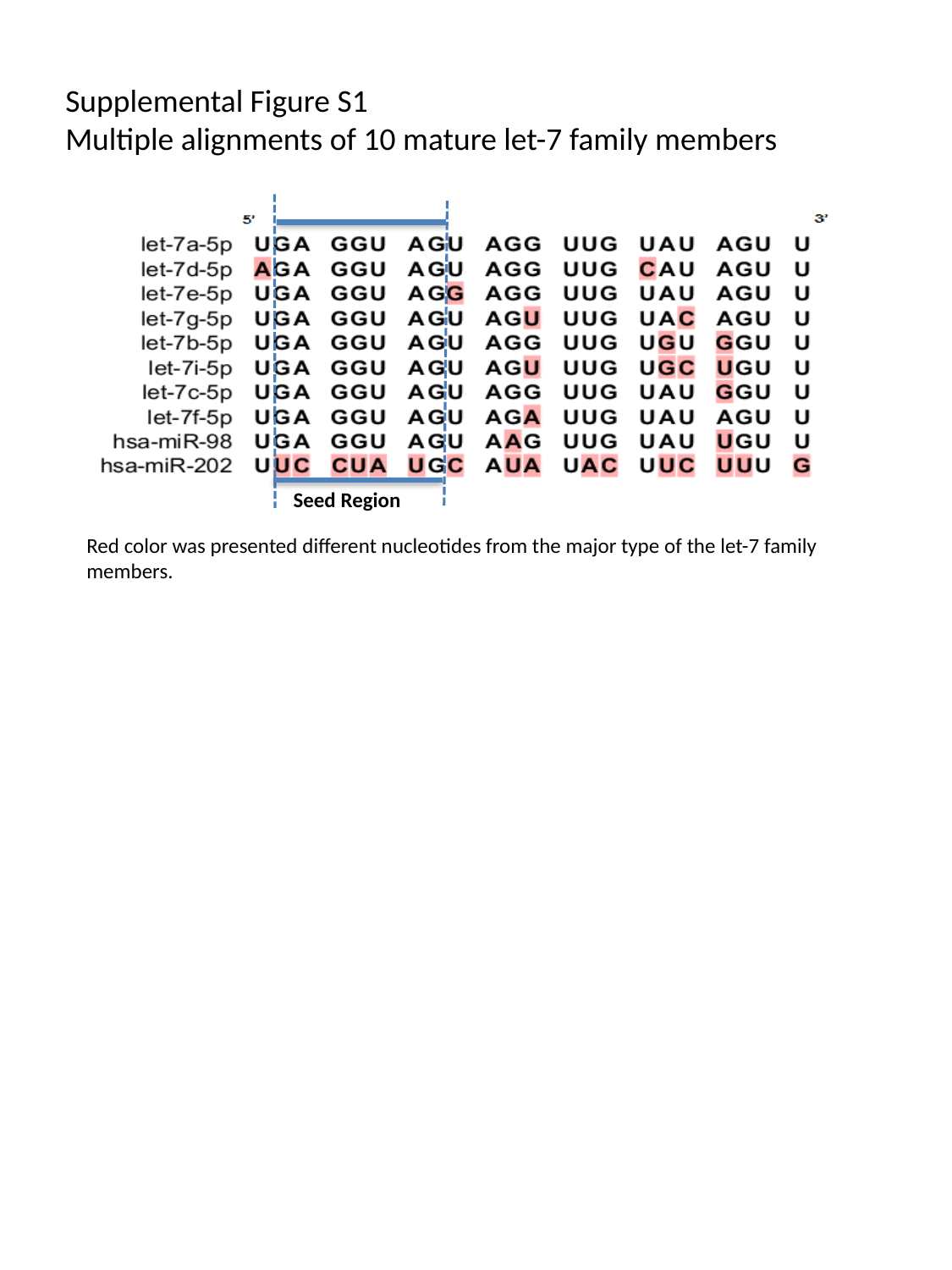

Supplemental Figure S1
Multiple alignments of 10 mature let-7 family members
Seed Region
Red color was presented different nucleotides from the major type of the let-7 family members.

## Slide 2
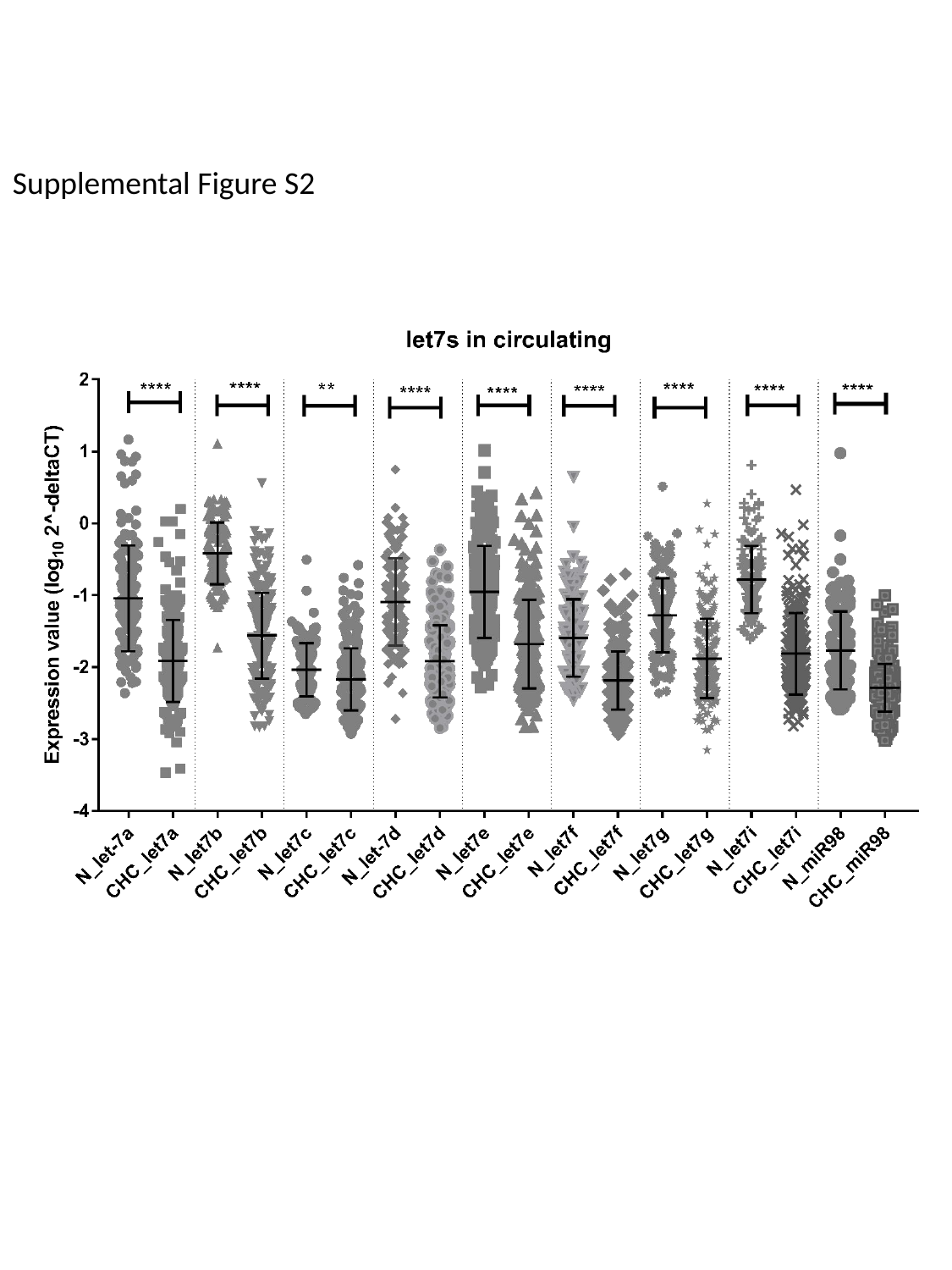

Supplemental Figure S2

## Slide 3
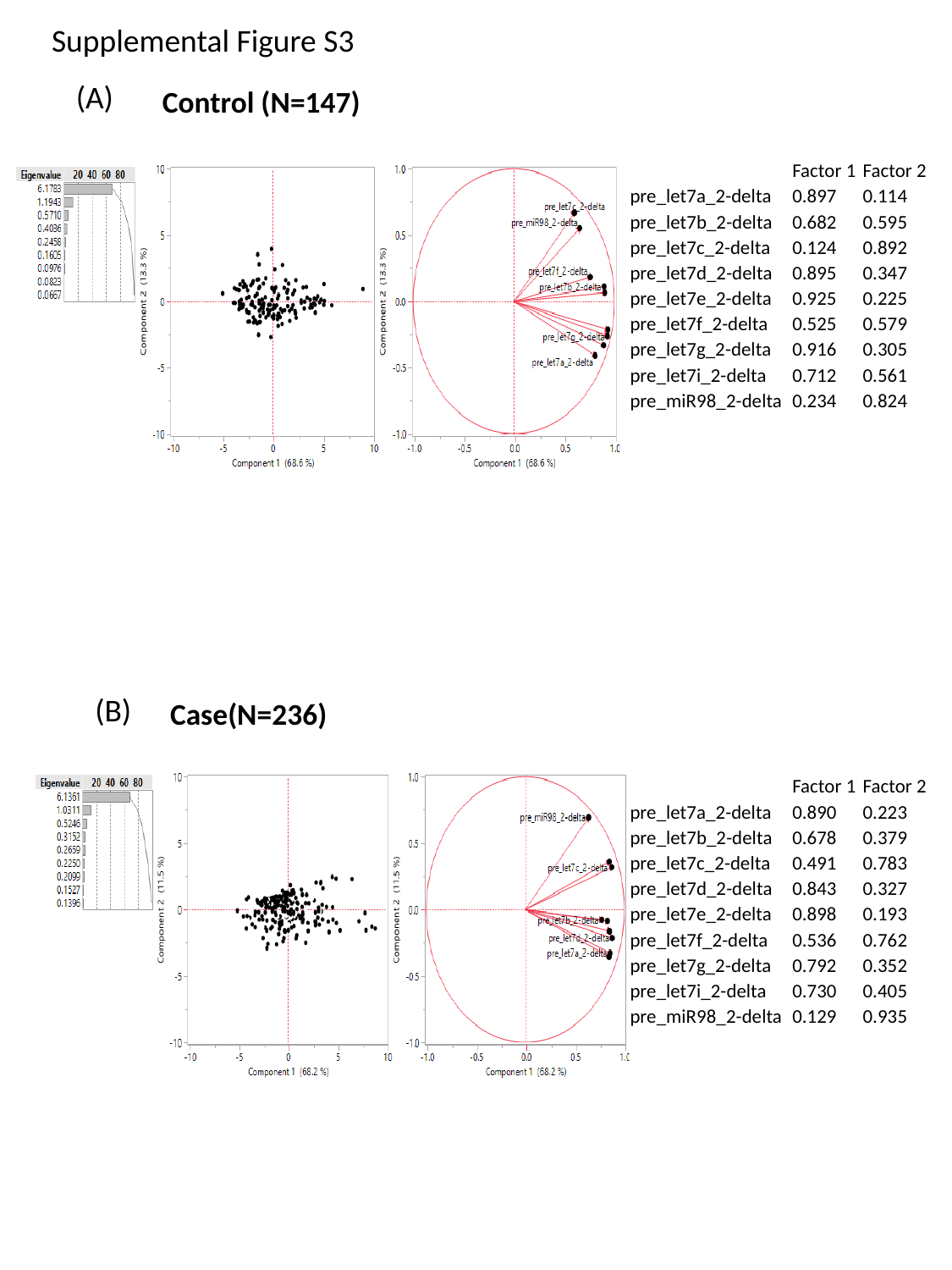

Supplemental Figure S3
(A)
Control (N=147)
| | Factor 1 | Factor 2 |
| --- | --- | --- |
| pre\_let7a\_2-delta | 0.897 | 0.114 |
| pre\_let7b\_2-delta | 0.682 | 0.595 |
| pre\_let7c\_2-delta | 0.124 | 0.892 |
| pre\_let7d\_2-delta | 0.895 | 0.347 |
| pre\_let7e\_2-delta | 0.925 | 0.225 |
| pre\_let7f\_2-delta | 0.525 | 0.579 |
| pre\_let7g\_2-delta | 0.916 | 0.305 |
| pre\_let7i\_2-delta | 0.712 | 0.561 |
| pre\_miR98\_2-delta | 0.234 | 0.824 |
(B)
 Case(N=236)
| | Factor 1 | Factor 2 |
| --- | --- | --- |
| pre\_let7a\_2-delta | 0.890 | 0.223 |
| pre\_let7b\_2-delta | 0.678 | 0.379 |
| pre\_let7c\_2-delta | 0.491 | 0.783 |
| pre\_let7d\_2-delta | 0.843 | 0.327 |
| pre\_let7e\_2-delta | 0.898 | 0.193 |
| pre\_let7f\_2-delta | 0.536 | 0.762 |
| pre\_let7g\_2-delta | 0.792 | 0.352 |
| pre\_let7i\_2-delta | 0.730 | 0.405 |
| pre\_miR98\_2-delta | 0.129 | 0.935 |

## Slide 4
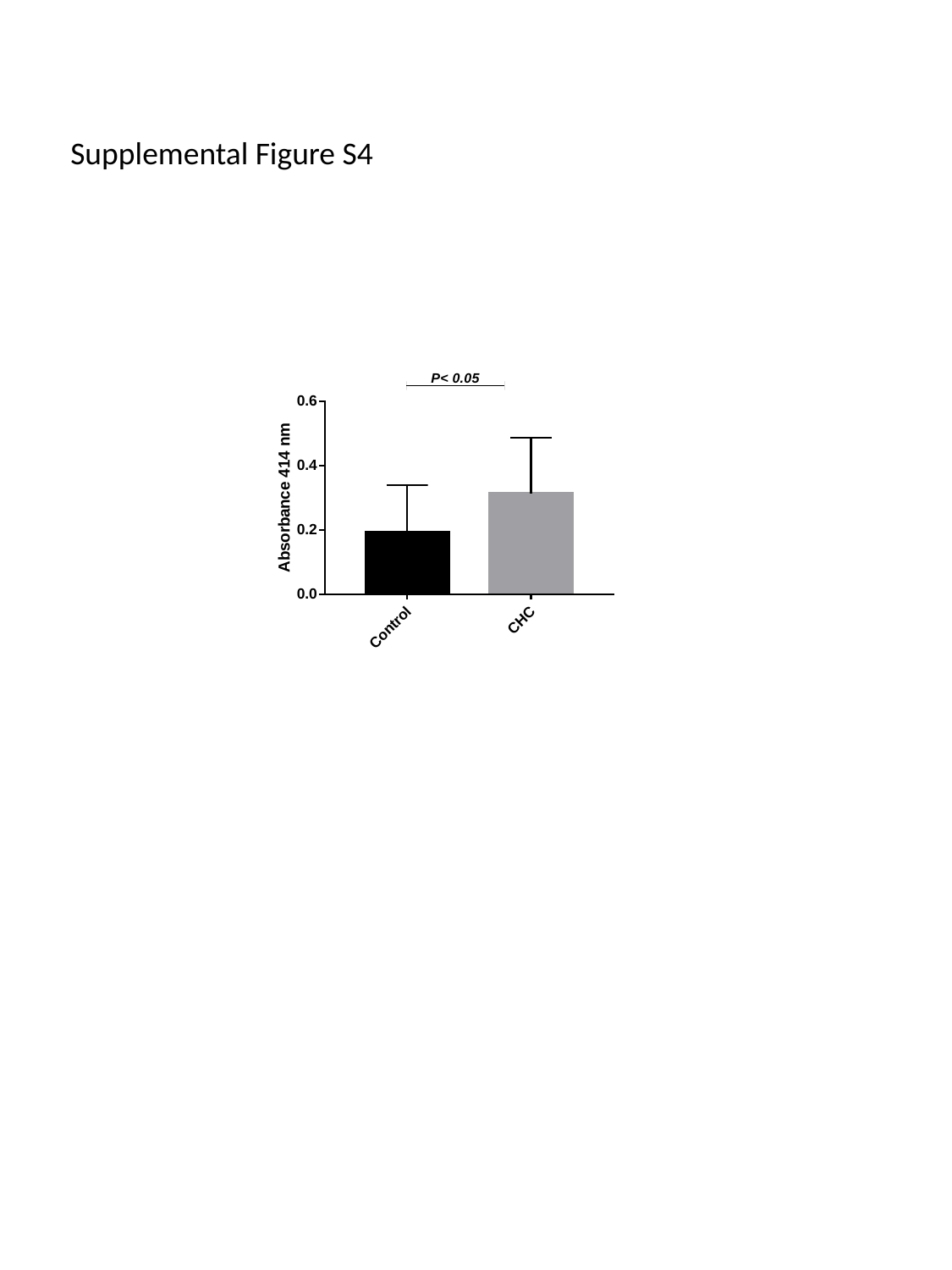

Supplemental Figure S4
